# Infection-induced chromatin modifications facilitate translocation of herpes simplex virus capsids to the inner nuclear membrane

**DOI:** 10.1371/journal.ppat.1010132

**Published:** 2021-12-15

**Authors:** Vesa Aho, Sami Salminen, Salla Mattola, Alka Gupta, Felix Flomm, Beate Sodeik, Jens B. Bosse, Maija Vihinen-Ranta

**Affiliations:** 1 Department of Biological and Environmental Science, Nanoscience Center, University of Jyväskylä, Jyväskylä, Finland; 2 HPI, Leibniz-Institute for Experimental Virology, Hamburg, Germany; 3 Centre for Structural Systems Biology, Hamburg, Germany; 4 Hannover Medical School, Institute of Virology, Hannover, Germany; 5 Cluster of Excellence RESIST (EXC 2155), Hannover Medical School, Hannover, Germany; University of Wisconsin-Madison, UNITED STATES

## Abstract

Herpes simplex virus capsids are assembled and packaged in the nucleus and move by diffusion through the nucleoplasm to the nuclear envelope for egress. Analyzing their motion provides conclusions not only on capsid transport but also on the properties of the nuclear environment during infection. We utilized live-cell imaging and single-particle tracking to characterize capsid motion relative to the host chromatin. The data indicate that as the chromatin was marginalized toward the nuclear envelope it presented a restrictive barrier to the capsids. However, later in infection this barrier became more permissive and the probability of capsids to enter the chromatin increased. Thus, although chromatin marginalization initially restricted capsid transport to the nuclear envelope, a structural reorganization of the chromatin counteracted that to promote capsid transport later. Analyses of capsid motion revealed that it was subdiffusive, and that the diffusion coefficients were lower in the chromatin than in regions lacking chromatin. In addition, the diffusion coefficient in both regions increased during infection. Throughout the infection, the capsids were never enriched at the nuclear envelope, which suggests that instead of nuclear export the transport through the chromatin is the rate-limiting step for the nuclear egress of capsids. This provides motivation for further studies by validating the importance of intranuclear transport to the life cycle of HSV-1.

## Author summary

Herpes simplex virus type 1 is a DNA virus that is studied intensively due to its high prevalence in humans, its ability to cause many diseases upon lytic replication or to evade the immune system in a latent state, and its potential use in oncolytic and immunotherapeutic applications. The nuclear replication of herpes simplex virus type 1 leads to the emergence of viral replication compartments together with relocalization and condensation of the host cell chromatin to the nuclear periphery. The reorganization of the host chromatin potentially hampers the intranuclear transport and nuclear egress of newly assembled progeny capsids. Nuclear capsids move by diffusion prior to their viral nuclear egress complex-mediated exit through the nuclear envelope. However, it is not understood how the capsids travel through the chromatin network. Our results show that infection-induced changes in chromatin architecture enhance nuclear diffusion of capsids at late infection, and that this transport is the rate-limiting step in the nuclear egress of the virus. Understanding the kinetics of capsid translocation in the nucleus is of fundamental interest in the field of DNA virus research because it explains how viruses are able to move in the chromatin before their nuclear egress.

## Introduction

Enveloped herpesviruses are widespread and important human pathogens. Herpes simplex virus 1 (HSV-1), a causative agent of the common cold sores, is a virus with a DNA-containing icosahedral capsid of ~125 nm in diameter [1]. Upon entering host cells, the viral capsids must travel to the nuclear envelope for docking to the nuclear pores where they expel their genomes into the nucleus [2–4]. The early studies of HSV-1 capsid motion in the cytoplasm elucidated that the motion to the nuclear envelope occurs via microtubules [5–7]. After capsid association with the nuclear pore complex, the viral genomes are injected into the nucleus and the formation of viral replication compartments (VRCs) is initiated. VRCs are dedicated sites for virus genome replication, and recent studies have revealed that each VRC originates from one individual viral genome [8–12]. As the infection progresses, the VRCs expand and the likelihood of their fusion and formation of enlarged compartments increases [4,13]. VRCs become enriched with capsid proteins and capsids as the infection progresses [14,15], but whether capsid assembly and packaging occur within the VRCs or whether the capsids accumulate at them later is not clear. However, capsids need to travel from the VRCs to the nuclear border for egress through the nuclear envelope into the cytosol [16–18]. The relatively large capsids with a diameter of 125 nm move through the nucleoplasm by diffusion [19,20]. Due to their large size, the capsids cannot pass through the nuclear pores, but instead bud through the nuclear envelope utilizing the nuclear egress complex formed by viral proteins UL31 and UL34 [21–23]. This process favors nucleocapsids, and most of the cytoplasmic capsids are DNA containing even though in the nucleus they are the in the minority [24,25]. After envelopment of the capsid at the inner nuclear membrane and subsequent envelope fusion at the outer membrane, capsids are released into the cytosol and transported to the *trans*-Golgi network or an endosomal compartment for final envelopment before they are released from the cell by exocytosis [18,26,27].

While the dynamics of cytoplasmic HSV-1 capsids have been investigated in detail [28], nuclear diffusion and its restriction by the chromatin network has been considered only recently [19,20,29,30]. Herpesvirus capsids are relatively large particles for intracellular transport; microinjected dextran beads of 110 nm, similar in size to HSV-1 capsids, are immobile in the nuclei of non-infected cells [31]. However, recent studies have shown that HSV-1 capsids diffuse inside the interchromatin domains that enlarge during the infection [20]. As the infection progresses further, the growing VRCs containing most of the progeny capsids displace the host chromatin toward the nuclear envelope and the center of the nucleus becomes mostly devoid of chromatin [29,30,32]. Previous studies have implied that VRCs are molecularly crowded and dynamic structures with temporal changes in their biomolecular composition [10,33–35]. As the chromatin is marginalized to the nuclear periphery, the chromatin-empty regions located in the central parts of the nucleus remain enriched with virus capsids. The displaced chromatin accumulates underneath the nuclear envelope as a layer that is most concentrated around 8 hours post infection (hpi), but subsequently low-density regions and channels that traverse the marginalized chromatin appear, which likely guide the capsids to the nuclear envelope [30,36].

Although the virus-induced modulation of the chromatin architecture and the capsid kinetics are increasingly understood, the mechanisms of capsids motion inside the infection-modified chromatin network have not been characterized. Herein, we have tracked fluorescently tagged HSV-1 capsids in living cells and correlated the intranuclear capsid localization and motion with the local chromatin environment. The progeny capsids were concentrated in the VRC-incorporating chromatin-empty regions, and the size of these regions increased as the infection progressed. The capsid density in the chromatin-empty regions was always higher than in the chromatin regions. Even though there was almost twofold increase in the number of detected capsids from 4 to 8 hpi, the capsid density in the chromatin increased only later at 12 hpi. Moreover, our analyses of the trajectories of individual capsids indicated that they were more likely to enter chromatin regions at 12 hpi than at 8 hpi. These data suggest that the chromatin restricted capsid motion as it was marginalized at 8 hpi. Later the permeability of chromatin was increased, which would facilitate the capsids to access the nuclear envelope. We further observed that capsids diffused faster in the chromatin-empty than in the chromatin regions, and that in both the diffusion rate increased as the infection progressed. At no time point was there accumulation of capsids at the nuclear envelope, which indicates that capsid translocation through the chromatin is a slower process than the transport through the nuclear envelope. Visualization of infected cells with electron microscopy confirmed the low density of capsids at the nuclear envelope and further showed that the number of DNA-containing capsids in the nucleus was relatively low, which may be an additional reason for the low density of capsids at the nuclear envelope since the DNA-containing capsids are preferentially retained there for budding. Altogether, these results imply that the nuclear environment undergoes changes during HSV-1 infection that modulate intranuclear motion and localization of capsids.

## Results

To examine the motion of capsids inside the infection-modified chromatin environment, we infected Vero cells with HSV-1 VP26-mCherry and stained their nuclei with Hoechst 33342. Time-dependent changes in the chromatin distribution, capsid localization and capsid dynamics were studied at 4, 8 and 12 hpi using spinning disk fluorescence microscopy. The analyses were conducted by comparing capsid localization and motion with local chromatin concentration.

### Distribution of chromatin and nuclear viral capsids

**Fig 1:**
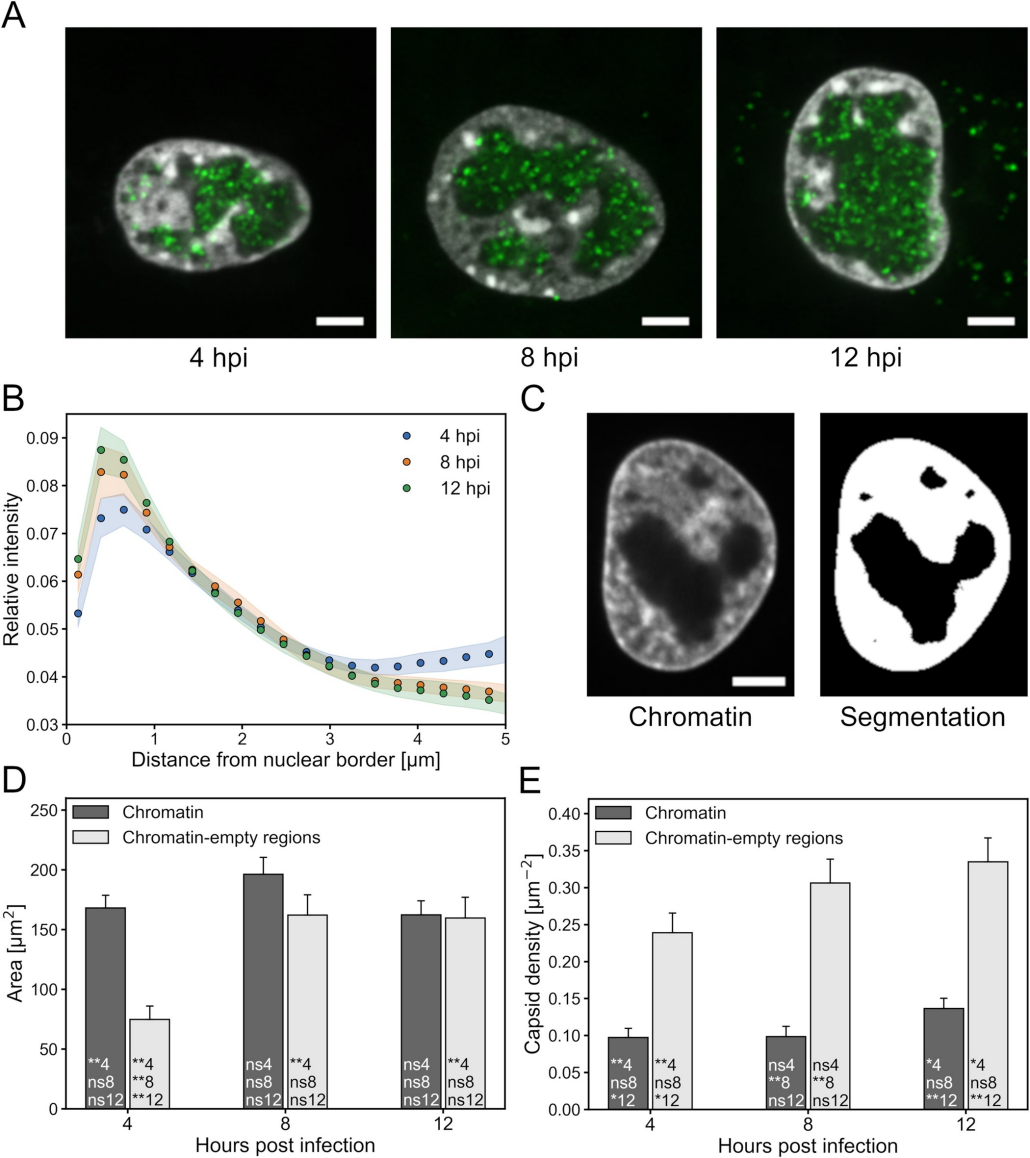
Capsids are concentrated in the chromatin-empty regions that grow during the infection. (A) Spinning disk microscopy images of capsid distribution in Vero cells at 4, 8 and 12 hpi. Overlays of Hoechst-labeled chromatin (gray) and fluorescent capsid protein VP26-mCherry (green) are shown. (B) The mean relative intensity of Hoechst-stained nuclear chromatin as a function of the distance from the nuclear envelope at 4 (blue), 8 (orange) and 12 hpi (green). The shaded areas around the data points represent the SEM. (C) Hoechst-stained chromatin (gray) in an infected cell nucleus at 8 hpi and its automatic segmentation into chromatin (white) and chromatin-empty regions (black). (D) The mean area of segmented chromatin (dark gray) and chromatin-empty regions (light gray) and (E) the mean density of capsids in chromatin and chromatin-empty regions. The error bars show the SEM. Statistical significances were determined using Student’s t-test. The significance values shown inside the bars are denoted as ** (p<0.01), * (p<0.05) or ns (not significant). The number after the significance symbol indicates the infection time point that the value was compared with. Values were compared for the same region at different time points (indicated by a different time code than the time point of the bar) and for the different regions only within each time point (indicated by the same time code as the time point of the bar). For every time point the sample size was 28 cells. The scale bars represent 5 μm.

The microscopy images indicated that the regions with low-intensity chromatin staining were located at the central parts of the nuclei and contained most of the viral capsids (Fig 1A). We also verified by a separate immunolabeling experiment that the low intensity region consisted mostly of VRC (S1 Fig). To quantify the infection-induced rearrangement of chromatin, we segmented the nuclei based on the DNA staining and examined the chromatin distribution with respect to the nuclear border (Fig 1B). The association of the chromatin border with the nuclear border was justified by the fact that chromatin is known to be separated from the nuclear envelope by the nuclear lamina, whose thickness is below the resolution of light microscopy (S2 Fig). The comparison of chromatin localization at various time points showed that the relative amount of DNA signal near the nuclear border increased from 4 to 8 hpi as the amount in the central parts decreased. From 8 to 12 hpi, the signal had shifted only slightly towards the nuclear border.

Both the chromatin and the chromatin-empty regions are important regarding capsid transport. The chromatin-empty VRCs contain most of the newly assembled progeny capsids at any given time, and these capsids need to traverse the marginalized chromatin to get access to the nuclear envelope for egress into the cytosol. To gain more insight into the interplay of capsids and chromatin structure, we examined the capsid localization and later capsid motion in these regions by automatically segmenting the nucleus of each cell into chromatin and chromatin-empty regions (Figs 1C and S3). Throughout the infection, the chromatin cross-sectional area remained almost unaltered (Fig 1D). This also suggested that the segmentation of the chromatin regions was quite accurate. Importantly, the cross-sectional area of chromatin-empty regions increased more than twofold from 4 to 8 hpi, but then did not change significantly from 8 to 12 hpi.

To further assess the intranuclear localization of the HSV-1 capsids, the images were segmented and capsid coordinates recorded. The analysis revealed that the capsid density in the chromatin-empty regions increased from 4 to 12 hpi (Fig 1E). Even though the total number of capsids increased from 4 to 8 hpi, the density of capsids in the chromatin did not increase during this time. However, at 12 hpi an increase was detected. At every time point the capsid density was much higher in the chromatin-empty regions than in the chromatin regions. The capsid density ratio between the regions was 2.6 ± 0.4, 3.9 ± 0.5 and 2.8 ± 0.4 at 4, 8 and 12 hpi respectively.

In summary, these results show that the chromatin-empty regions, which mostly correspond to the VRCs, grow as the chromatin is displaced toward the nuclear periphery. The capsid density was much higher in the chromatin-empty than in the chromatin regions from 4 to 12 hpi, which suggests that capsid motion was restricted by the chromatin regions. The capsid density ratio between chromatin and chromatin empty regions was highest at 8 hpi, indicating that chromatin was less accessible to capsids at this time point. At 12 hpi the chromatin accessibility was partially recovered.

### Dynamics of capsids in the nucleus

The intranuclear mobility is physically restricted by local chromatin structures [37–40]. To elucidate how infection-induced changes in the nuclear environment affect capsid motion, we next studied the characteristics of capsid motion both in the chromatin and chromatin-empty regions by recording 400 images taken with 0.1 s intervals of cells infected with HSV-1 VP26-mCherry and stained for DNA (S1 Movie).

**Fig 2:**
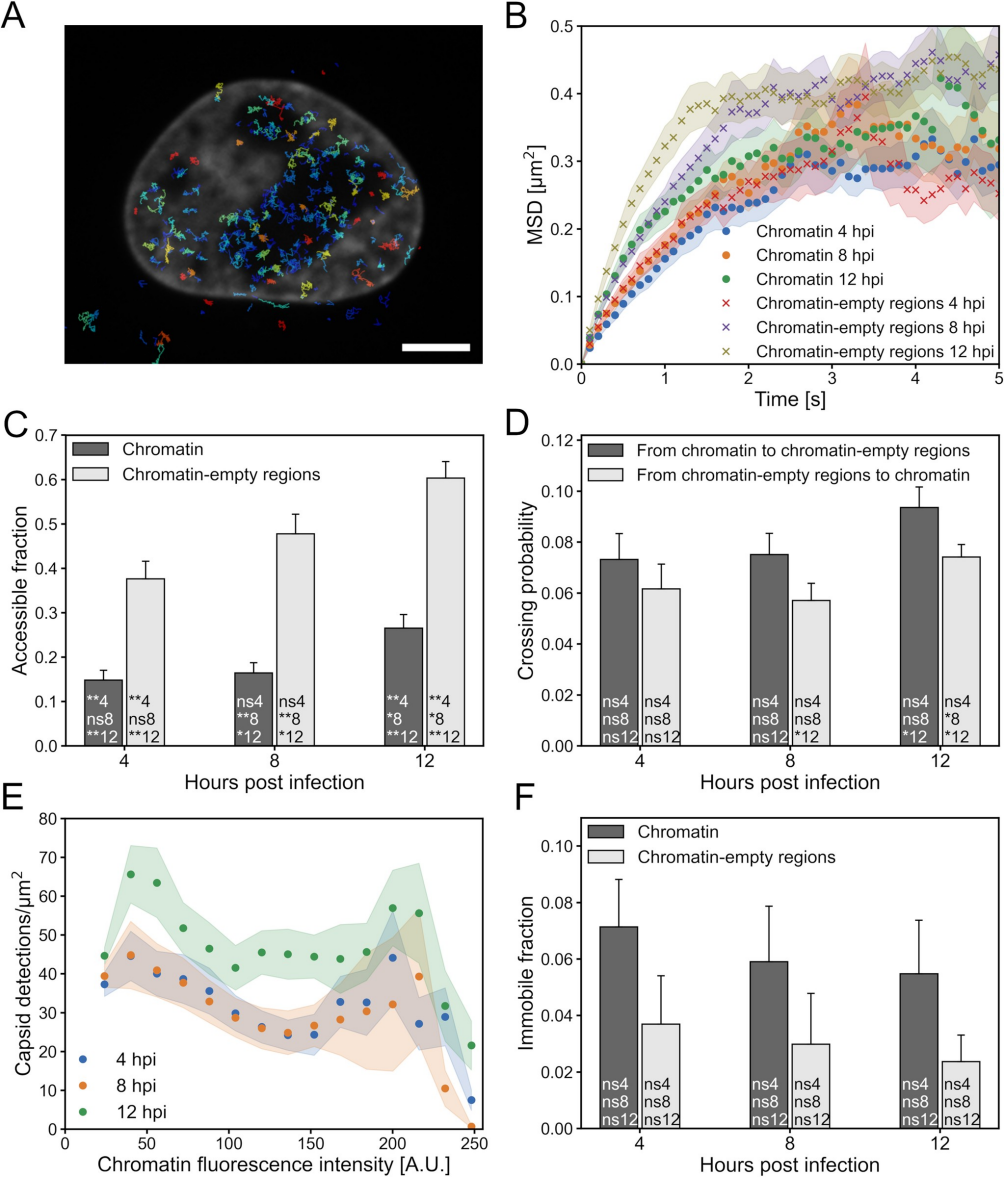
Capsid dynamics depend on the chromatin environment and on the infection phase. (A) Capsid tracks during 10 seconds in an HSV-1 infected cell. Only tracks that were at least 5 frames (0.5 s) long are shown. Cytoplasmic tracks are also shown, but they were excluded from the analyses. The stained chromatin is shown in gray. (B) The mean squared displacement (MSD) of nuclear capsids in chromatin and chromatin-empty regions as a function of time. (C) The mean fraction of chromatin and chromatin-empty regions visited by capsids during 40 s. (D) The mean probability of a capsid starting on one side of the border of chromatin regions to move to the other side of a border during a capsid track. (E) The mean number of capsids detected in chromatin during 40 s as a function of chromatin density. The number of detections was normalized by the size of the detection area. (F) The ratio of immobile particles to the number of all particles in chromatin and chromatin-empty regions. The shaded areas around the data points and the error bars show the SEM. Statistical significances were determined using Student’s t-test. The significance values shown inside the bars are denoted as ** (p<0.01), * (p<0.05) or ns (not significant). The number after the significance symbol indicates the infection time point that the value was compared with. Values were compared for the same region at different time points (indicated by a different time code than the time point of the bar) and for the different regions only within each time point (indicated by the same time code as the time point of the bar). For every time point the sample size was 28 cells. The scale bar represents 5 μm.

The capsids were detected in each image, and their tracks were documented based on the individual capsid coordinates. The capsids seemed to move primarily in the low chromatin density regions, but a smaller fraction moved also within the denser chromatin regions at all three time points (Fig 2A). We examined capsid motion by correlating the extracted capsid coordinates with the segmented chromatin regions. We calculated the mean squared displacement (MSD) of capsids to determine how the chromatin affected the capsid mobilities during the course of the infection (Fig 2B). The curves had the characteristic shapes of confined diffusion, showing an initial rapid increase in the MSD and a final saturation due to particles being unable to penetrate boundaries of the confining regions. The saturation started to affect curves quite early, but at a short time scale the curves could be fitted with an equation for anomalous diffusion, *D* = 4*Dt*^*α*^, where *D* is the diffusion coefficient and *α* is the anomalous exponent (S4 Fig). The obtained diffusion coefficients and anomalous exponents at the time scale of 0–1.0 s are summarized in Table 1.

**Table 1:** The diffusion coefficients and anomalous exponents of HSV-1 capsids in the chromatin and chromatin-empty regions of the HSV-1-infected cell nuclei at 4, 8 and 12 hpi. The mean ± SEM are shown.

		4 hpi	8 hpi	12 hpi
Chromatin	*D* [*μ*m^2^/s]	0.044 ± 0.005	0.053 ± 0.005	0.070 ± 0.007
	*α*	0.90 ± 0.04	0.86 ± 0.03	0.83 ± 0.03
Chromatin-empty regions	*D* [*μ*m^2^/s]	0.047 ± 0.006	0.063 ± 0.005	0.085 ± 0.008
	*α*	0.69 ± 0.03	0.78 ± 0.05	0.75 ± 0.02

The motion of capsids appeared to be subdiffusive (*α* < 1): the mean anomalous exponent was between 0.69 and 0.90 in both regions at every measured infection time point. At any given time point, the diffusion coefficient was higher in the chromatin-empty than in the chromatin regions, and the ratio of the diffusion coefficients between the regions was lowest at 4 hpi and highest at 8 hpi. Interestingly, both in the chromatin and chromatin-empty regions the diffusion coefficient increased as the infection progressed. In chromatin the difference between 4 and 8 hpi was not very large, but at 12 hpi a bigger change occurred. In the chromatin-empty regions there was a steadier increase from 4 to 12 hpi.

The MSD value where the curve saturates can give indication of the size of the compartments where the particles diffuse in. The actual saturation values were difficult to determine due to large uncertainties of the curves (which probably result from large variations in the sizes of confining regions and from the lower number of tracks extending to longer times). The size of the diffusion compartments seemed to be, however, from about 1.0 μm to 1.4 μm when analyzed using equation $d_{c}\;=2\sqrt{MSD_{sat}}+d_{HSV}$, where *MSD*_*sat*_ is the saturation value of the *MSD* and *d*_*HSV*_ is the capsid diameter of 125 nm [20].

Next, we analyzed the fraction of pixels in the chromatin and chromatin-empty regions that had a capsid visiting them during the recorded time series (Fig 2C). The fraction in the chromatin-empty regions increased steadily from 4 to 12 hpi, but the fraction in chromatin remained almost constant from 4 to 8 hpi, after which it clearly increased at 12 hpi.

To examine the motion of capsids between the nuclear regions, we analyzed the probability of a capsid starting on either side near the border between chromatin and chromatin-empty regions and moving to the other side (Fig 2D). The probability to cross the interface from chromatin-empty regions to chromatin decreased from 4 to 8 hpi, but this decrease was not statistically significant (p>0.05). However, from 8 to 12 hpi, there was a significant increase in the crossing probability (p<0.05). This is in line with the chromatin accessibility data described above and with our previous study indicating that marginalized chromatin is denser at 8 hpi after which the density decreases at 12 hpi [30].

As there was a clear difference in the capsid density between chromatin and the chromatin-empty regions, we analyzed if the chromatin density within the segmented chromatin affected the occurrence of capsids at those locations. The low-density pixels had a high probability of capsid detection, but surprisingly the effect was not very strong (Fig 2E). Even at high chromatin densities there was a relatively high probability to find capsids in those regions. The total size of the regions with such high chromatin fluorescence intensity values was, however, very small. Finally, to gain deeper understanding on restrictions to capsid movement, the number and locations of immobile capsids were analyzed (Fig 2F). At every infection time point the fraction of immobile capsids in chromatin was higher than in chromatin-empty regions. However, the variations were so large that no statistically significant differences could be established.

Taken together, the difference in the crossing probability between chromatin and chromatin-empty regions at 8 and 12 hpi is consistent with the notion that capsid motion is restricted by chromatin. However, the increase in the capsid diffusion coefficient in chromatin during the infection suggests that density of chromatin decreases as the infection progresses. The same appears to be true for the chromatin-empty regions. It should be also noted that the transport probability between chromatin and chromatin-empty regions was affected by the infection-induced changes.

### Capsid motion relative to the nuclear border

**Fig 3:**
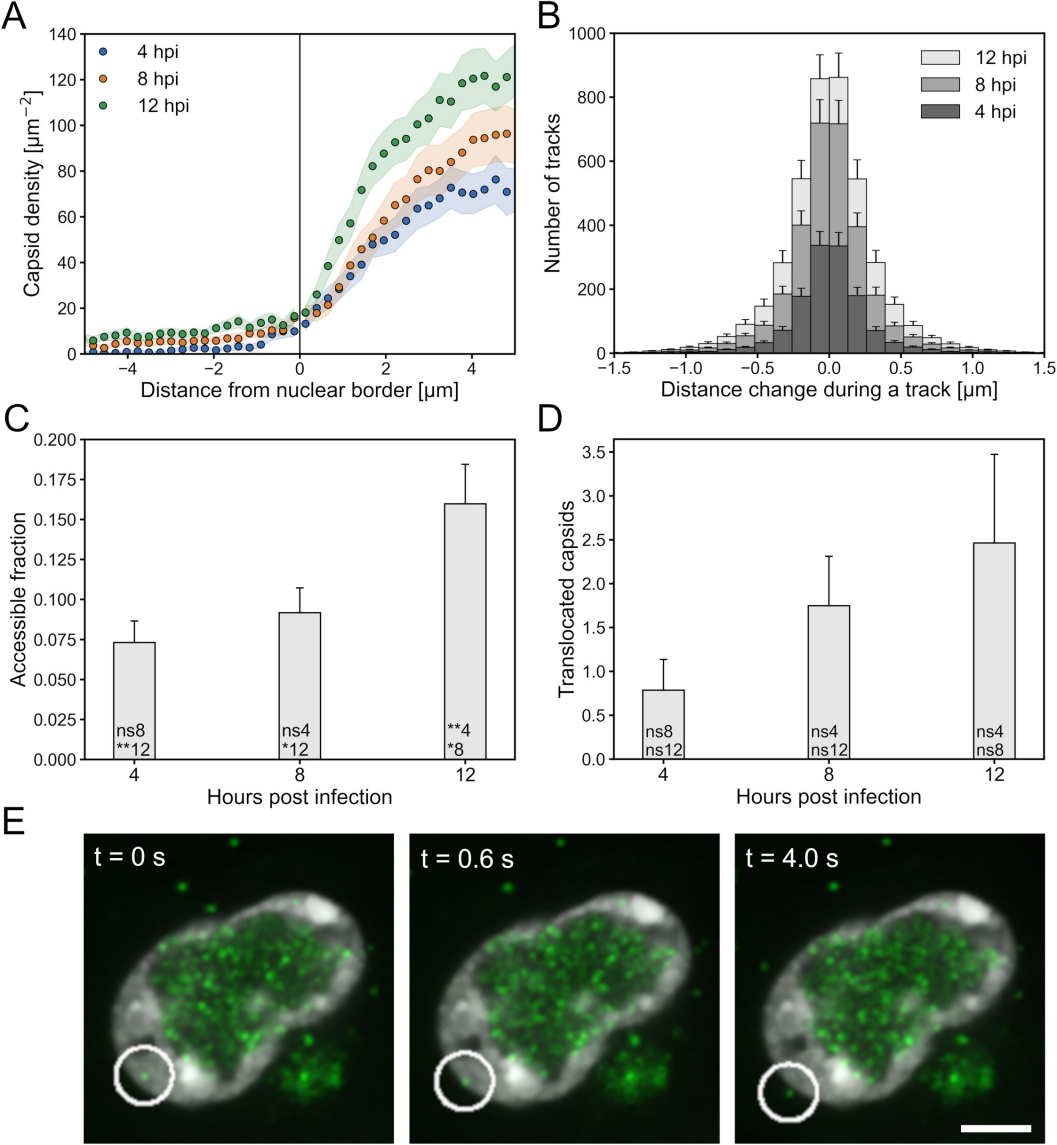
Capsids do not accumulate at the nuclear envelope. (A) The mean density of capsids as a function of distance from the nuclear envelope summed over the time series. The negative *x*-axis values show the distance to the cytoplasmic side and positive *x*-axis values the distance to the nucleoplasmic side of the nuclear border. (B) The change in the shortest distance to the nuclear border during a capsid track. On the positive *x*-axis the capsid moves away from the nuclear border during a track and on the negative *x*-axis toward it. (C) The fraction of the area within 1 μm from the outer edge of the chromatin visited by capsids during 40 s. (D) The mean number of capsids that translocated from within the chromatin to outside of it at the edge of the nucleus. (E) An image series showing a capsid approaching the edge of the chromatin at the border of the nucleus and passing through it. The error bars show the SEM. The significance values shown inside the bars are denoted as ** (p<0.01), * (p<0.05) or ns (not significant). The number after the significance symbol indicates the infection time point that the value was compared with. For every time point the sample size was 28 cells. The scale bar represents 5 μm.

In searching for factors that might modulate capsid egress, we examined capsid localization and motion relative to the nuclear border. To determine whether egressing capsids were retained at the nuclear envelope, we determined the density of capsids as a function of distance from the nuclear border on both the nuclear and cytoplasmic sides of it (Fig 3A). Interestingly, we did not detect any capsid accumulation near the nucleus-cytoplasm interface. Next, we tested whether capsid motion was directed toward the nuclear envelope by comparing the distance to the nuclear border at the beginning and the end of each capsid track. At each time point, the histograms were quite symmetrical with respect to whether the capsids were moving toward or away from the nuclear border, indicating that there was no preferred direction with respect to the nuclear envelope (Fig 3B). To characterize the capsid movements toward the nuclear envelope, we calculated the proportion of the region within 1 μm from the outer edge of the chromatin that had a capsid visiting them during the imaged time course. At 12 hpi, the capsids were able to access a proportionally larger region than at 4 or 8 hpi (Fig 3C). Finally, we determined the number of capsids that had started their tracks within a chromatin region and were able to leave it at the border of the nucleus (Fig 3D and 3E). Our data show that the number of capsids traveling through the chromatin regions increased steadily. At 12 hpi, the number was highest, but there was also a large variation between cells.

### Electron microscopy analysis of HSV-1 capsids in the nucleus

**Fig 4:**
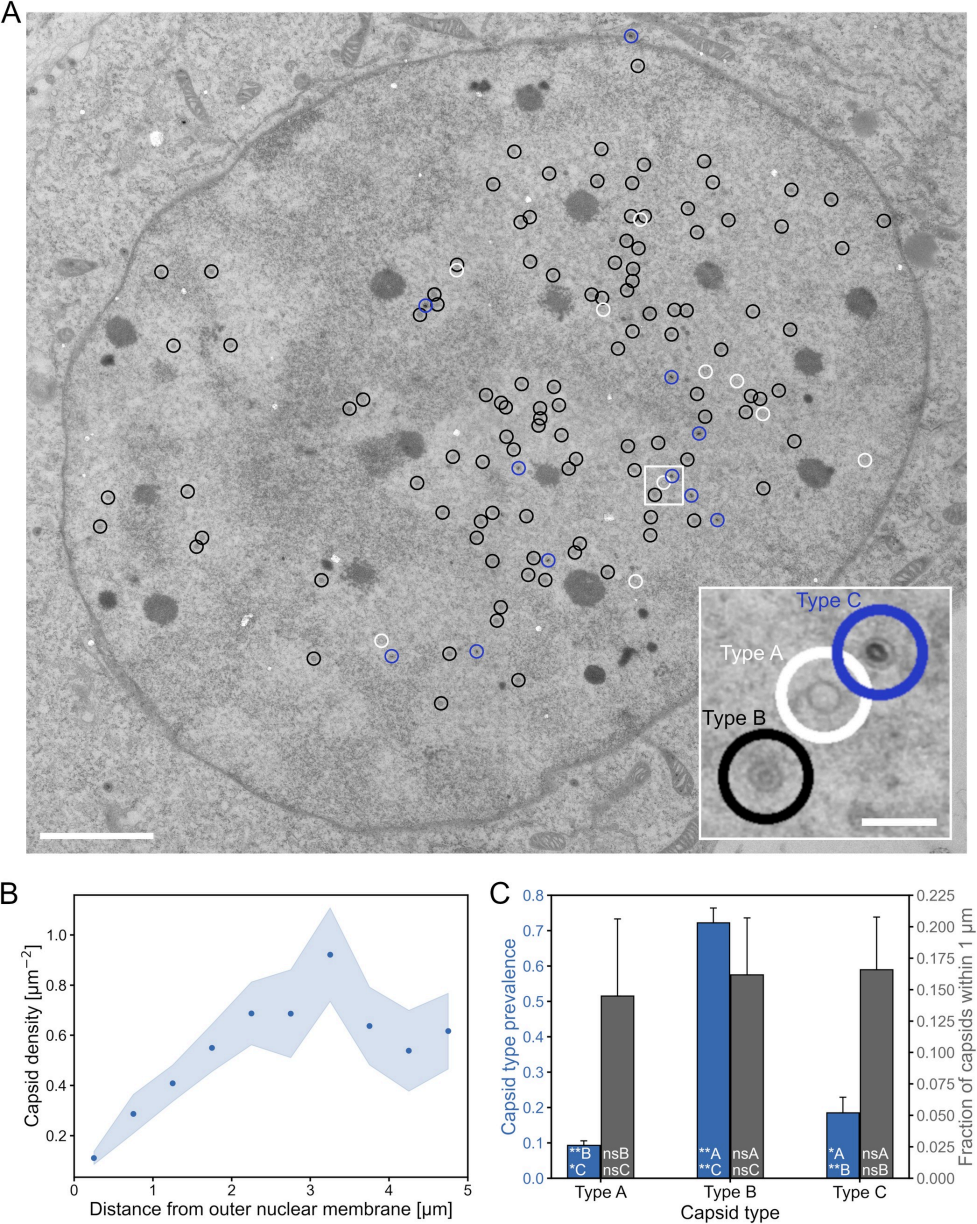
The capsid type does not affect its ability to traverse marginalized chromatin. (A) A transmission electron microscope image of a cell infected 12 h prior to sample fixation. The capsids have been labeled as type A (empty capsids, marked with white circles), type B (capsids containing the protein scaffold, black circles) and type C (DNA-containing nucleocapsids, blue circles). The area marked with a white square is magnified at the lower right corner of the image, showing each of the capsid types. The scale bar represents 2 μm (200 nm in the magnified region). (B) The combined density of every capsid type as a function of the distance from the outer nuclear membrane. (C) The number of each capsid type divided by the number of all capsids (blue), and the fraction of each capsid type that is located within 1 μm from the nuclear envelope (gray). The shaded regions and error bars show the standard error of the mean. The significance values shown inside the bars are denoted as ** (p<0.01), * (p<0.05) or ns (not significant). The letter after the significance symbol indicates the capsid type that the value was compared with. The sample size was *N* = 11 cells.

To verify the low density of capsids at the nuclear envelope and to see if the localization of capsids in the nucleus depends on their stage of DNA packaging, we used electron microscopy to image cells infected 12 h before sample fixation. Visual observation revealed that relatively few capsids were located close to the nuclear envelope or were in the process of budding through it. In electron microscopy micrographs three types of capsids can be distinguished: empty capsids (type A), capsids that contain the inner protein scaffold (type B) and capsids that contain the viral genome (type C). We manually annotated capsids by their type based on their appearance (Fig 4A). Analysis of capsid density as a function of distance from the outer nuclear membrane verified that the number of capsids was lowest near the nuclear membrane (Fig 4B). We calculated the prevalence of each capsid type in the nucleus (Fig 4C), and the B type was the most common with the value of 72 ± 4%. The portion of A capsids was 9.3 ± 1.2% and C capsids 19 ± 4%. Between 14% and 17% of each capsid type was located within 1 μm from the outer nuclear membrane, and there was no statistically significant difference between the capsid types (Fig 4C).

Altogether, this shows that the DNA-containing capsids are not more likely to cross the marginalized host chromatin than the other capsids. The transport through the chromatin is not a selective process and the selection of capsids for nuclear egress takes place later at the nuclear envelope.

## Discussion

The mechanisms that regulate the intranuclear transport of newly assembled, progeny HSV-1 capsids to the nuclear envelope are not well understood. It is known that capsids traverse the nucleoplasm by a diffusion-based mechanism, but it is not clear how the presence of intranuclear structures such as chromatin regions or VRCs affect this motion. Herein, single-particle tracking of capsids in the nuclei of living cells with labeled chromatin allowed us to detect capsid motion with respect to the chromatin environment.

The establishment of nuclear HSV-1 VRCs is accompanied by a marginalization of the host cell chromatin to the nuclear periphery [29,32]. Our analysis of the chromatin distribution as the HSV-1 infection progressed showed that this chromatin relocalization correlated with a concomitant increase in the sizes of chromatin-empty regions and the nucleus. As more capsids were formed from 4 to 8 hpi, the density of capsids increased in the chromatin-empty regions but not in the chromatin regions. However, at 12 hpi the capsid density increased in the chromatin regions. Moreover, the ratio of the capsid density in the chromatin-empty regions to the capsid density in the chromatin regions was highest at 8 hpi, which suggests that chromatin was less accessible to capsids at this intermediate time point. This temporal decrease and subsequent increase in chromatin accessibility is consistent with our previous expansion microscopy findings that the proportion of interchromatin spaces in the marginalized chromatin decreases from 4 to 8 hpi but increases again at 12 hpi [30]. Our results suggest that infection-induced changes in the chromatin accessibility, particularly the chromatin redistribution induced by the VRC expansion, and the increase of interchromatin spaces later in infection, may play a significant role in accessibility of the chromatin to viral capsids, and thereby influence the dynamics of the HSV-1 nuclear egress during the infection.

During interphase, the molecularly crowded nuclear environment with the chromatin network, subnuclear compartments and many proteins limits diffusion in the nucleoplasm [41,42]. Particularly, the three-dimensional organization of the chromatin modulates mobility and interactions of nuclear macromolecules [43–45]. The structural hindering of motion by the chromatin is dependent on size of the macromolecule [37,46–48]. For example, diffusion of larger structures such as mRNPs particles with a diameter of up to 25 nm is hindered by dense chromatin regions [37,49,50]. Moreover, the molecularly crowded cytoplasm or the compact chromatin act as diffusion barriers for viral capsids, e.g. HSV-1 capsids with a diameter of 125 nm [20,29,51]. In infected cells, the infection-induced compaction and marginalization of chromatin poses an additional challenge for the nuclear egress of HSV-1 capsids [30,36]. Here, we discovered that the capsids exhibited subdiffusional behavior, and that the slopes of the MSD(t)-curves decreased before they saturated in the time scale of seconds. The mean anomalous exponents were between 0.69 and 0.90. Subdiffusion is a sign of obstructed diffusion, and it has been observed for example for the intranuclear motion of adeno-associated viruses (*α* = 0.6–0.9, [52]) and telomeres (*α* = 0.32, [53]). Our analysis of the fitted diffusion coefficients demonstrated that diffusion was always more rapid in the chromatin-empty than in the chromatin regions. This is consistent with our soft x-ray microscopy studies showing that the density of biomolecules is significantly lower in the VRCs than in tightly packed chromatin [29]. The ratio of diffusion coefficient in the chromatin-empty regions to the diffusion coefficient in the chromatin regions was highest at 8 hpi, after which it decreased when the infection proceeded to 12 hpi. The strong increase in the diffusion rate from 8 to 12 hpi agrees with our previous study showing that the fraction of chromatin-free regions increases substantially in the marginalized chromatin when visualized with expansion microscopy [30]. As the infection progressed, the diffusion coefficients increased both in chromatin and in chromatin-empty regions. It is noteworthy that the chromatin-empty regions containing enlarged VRCs with viral genomes, viral proteins and capsids could be a crowded environment. Such a molecular crowding could hinder diffusion, however, our measurements demonstrate that there was no reduction in the diffusion coefficient or the anomalous exponent in the VRCs. This suggests that the VRCs do not become more crowded as the infection proceeds.

Our mean squared displacement analysis shows that diffusion in chromatin and chromatin-empty region was restricted to regions between 1.0 μm and 1.4 μm in size, which is in accordance with a previous study [20]. Surprisingly, the differences among the confinement regions of chromatin and chromatin-empty regions were quite small. Furthermore, as the confinement regions were smaller than the nuclei or the VRCs, the capsid motion was not, at least in our analysis time scale, mainly restricted by the nuclear envelope or the marginalized chromatin but by smaller subnuclear structures. Moreover, our analyses indicate that the probability of particles to translocate from chromatin-empty to chromatin regions increased from 8 to 12 hpi, which is consistent with our finding that capsid movement became faster in chromatin. It is possible that the crossing probability is not only affected by chromatin compaction, but by interface effects at the border of the chromatin. It has been shown even for much smaller particles than HSV-1 capsids that the particles may diffuse quite freely within low and high chromatin density areas but the motion between the two regions is more restricted [54]. There was no clear correlation between the chromatin density and the capsid localization. In general, the probability of capsid detection was high in the low chromatin density regions, but the capsids were found also in the higher density regions. Possibly we were unable to detect smaller channels of low chromatin density with the ~200 nm resolution in the lateral direction and below 500 nm in the axial direction. Notably, there were some high chromatin density pixels that had a high probability of capsid detection in them, which can possibly result from capsids being trapped in those regions. The presence of immobile capsids correlated with the progress of infection, and the highest fraction of immobile capsids were detected at early times post infection. Immobile capsids were located both in the chromatin-empty and chromatin regions; their static positions could be due to steric hindrance by a high chromatin density or by genome packaging into newly pre-assembled capsid shells [55–58]. Although we have here analyzed capsid motion only with respect to chromatin, the capsid motion can also be restricted by other nuclear bodies. However, these structures are typically small compared to the size of the chromatin regions, and they are also located at the central parts of the nucleus and therefore not blocking the capsid access to the nuclear envelope. For this reason, their effect on the capsid transport to the nuclear envelope is probably much smaller compared to that of the chromatin.

We did not observe any substantial accumulation of capsids beneath the nuclear envelope. This indicates that the time scale of capsid transport to the nuclear envelope is so slow compared to the nuclear egress that an accumulation does not happen. Therefore capsid transport through the marginalized chromatin seems to be a bottleneck of the nuclear egress of HSV-1 progeny capsids. Moreover, we observed that the number of DNA-containing capsids was quite low constituting approximately only one fifth of all the nuclear capsids. Since they are the favored capsid types in the nuclear egress [25], their low number, combined with the transport barrier caused by the marginalized chromatin, leads to their low density at the nuclear envelope. The tendency of capsids to diffuse similarly toward or away from the nuclear envelope was observed, and it suggests that the diffusion of capsids toward the nuclear envelope was not facilitated. The number of capsids that travelled from within chromatin to outside of it at the nuclear border increased as the infection progressed, but we did not observe those capsids moving a substantial distance away from the nucleus. The HSV-1 budding process at the inner nuclear membrane mediated by the nuclear egress complex takes, at least *in vitro*, more than five minutes [26], and is therefore a slower process than we could monitor in our experiments.

Advances in the camera technology allow imaging fluorescently-labelled capsids with high sensitivity and temporal resolution. The quantum efficiency of the latest CMOS cameras exceeds 90%, so there is not much room for improvement in that respect. With future advances in super-resolution microscopy, capsid-chromatin interaction can potentially be studied with even finer detail. When studying a population of infected cells, the variability in the state of infection among the individual cells can be a problem. Although the cells in our assays were isogenic, the heterogenous cellular microenvironment and the population context variability lead to asynchronicity in the viral entry, the initiation of viral transcription, and all subsequent steps of the HSV-1 life cycle despite an inoculation with a high MOI [59,60]. One of the challenges in the future studies will be to control this heterogenous progression of HSV-1 infection among individual cells, or more likely to take this into account. With automated imaging and analyses a higher number of cells can be analyzed, leading to more accurate measurements even in the case of large variations between cells. It should also be noted that the cell type or tissue specific chromatin environment of the host likely has an impact on capsid mobility and progression of infection, which needs to be addressed in the future studies.

By a thorough investigation of intranuclear capsid motions, we discovered the changing properties of the nuclear environment during HSV-1 infection. The Brownian motion of particles is determined by the temperature and viscosity of the medium, the size and shape of the particles, and the surrounding obstacles restricting the motion. Since the temperature in our experiments was kept constant and the tracked capsids are of constant size, the only variables were the viscosity of the nucleoplasm and the effects of obstacles. Therefore, the analysis of intranuclear viral capsid dynamics is a powerful tool to indirectly probe the viscous and obstructive properties of the nucleoplasm. Examining the capsid motion in living cells opens a window into understanding of the rules that drive the nuclear transport of viral progeny capsids throughout the nonhomogeneous nuclear environment during their egress. We believe that our study provides new insights into intranuclear motion of capsids and introduces novel ideas for studies of intranuclear transport.

## Materials and methods

### Cells and viruses

For all the experiments Vero cells were grown at 37°C and 5% CO_2_. The cells were infected 4, 8 or 12 hours before measurements with HSV-1 VP26-mCherry virus (HSV1(17^+^)Lox-CheVP26) using multiplicity of infection of 5 [61–63]. In live cell experiments, the cells were stained 30 min before the measurements with 1 μg/ml of Hoechst 33342. The comparison of Hoechst and histone staining was done in a separate experiment by additionally labeling the cells by transfecting them with H2B-EGFP fusion protein plasmid using BacMam 2.0 CellLight H2B-GFP (40 particles per cell) (Invitrogen) one day before the infection.

For immunofluorescence studies of VRC or lamin B, the cells were fixed 4, 8 or 12 hours post infection with 4% paraformaldehyde for 10 min. Prior to immunolabeling, the fixed cells were permeabilized with 0.1% Triton X -100 in PBS supplemented with 0.5% BSA. VRCs were immunolabeled with mouse monoclonal antibody against HSV-1 ICP8 (Abcam, ab20194) and goat anti-mouse Alexa Fluor 546 (Abcam) secondary antibody. The nuclear lamina was immunolabeled with a rabbit polyclonal anti-Lamin B1 antibody (ab16048, Abcam, Cambridge, UK) and with a corresponding goat anti-rabbit Alexa Fluor 488 secondary antibody (Thermo Fisher Scientific, Waltham, MA, USA). DNA was stained with 1 μg/ml Hoechst 33342 (Thermo Fisher Scientific) for 12 min and washed twice with 1 x PBS á 3 min after which the samples were embedded with Prolong Glass antifade mountant (Thermo Fisher Scientific).

For EM experiments, an infected cell monolayer was immersed at 12 hpi in freshly prepared fixative solution (2% glutaraldehyde, 2% paraformaldehyde, 0.1 M sodium cacodylate buffer, pH 7.4) for 30 minutes at room temperature, followed by post-fixation with 1% OsO4 for 1 h on ice. The post fixed samples were dehydrated with graded series of ethanol, infiltrated and embedded in epon resin. Thin sections (approximately 80 nm) were cut from the hardened epon blocks and the sections at the nuclear level were post stained with aqueous uranyl acetate—lead citrate.

### Microscopy

The cells were imaged using a Nikon Eclipse Ti2 microscope equipped with a Yokagawa CSU-W1 spinning disk scanner, 100x Nikon 1.49 NA Apo-TIRF objective and an Andor iXon 888 EMCCD camera. The setup was equipped with 405, 488, 561, and 640 nm laser lines and corresponding filter sets. A single confocal plane was selected near the center of the nucleus, and imaging of mCherry-labeled capsids was done with the speed of 10 frames per second for the duration of 40 seconds. Hoechst distribution was recorded by taking one image before and, to verify that no significant movement of chromatin happened during the acquisition, one image after recording the time series of the mCherry channel. The pixel size was 130 nm. Hoechst was excited with a 405 nm diode laser and VP26-mCherry with a 561 nm DPSS laser. Life cell experiments were carried out in a humidified incubation chamber heated to 37°C and 5% CO2 controlled by a gas-mixer with cells were grown on Ibidi 35 mm glass-bottom dishes. The fixed samples were imaged with Leica SP8 X Falcon confocal microscope using HC PL APO 63x 1.4 NA oil immersion objective or with Nikon A1R confocal microscope with CFI Plan Apo VC 60XH 1.4 NA oil immersion objective. The electron microscopy samples were imaged with JEOL JEM -1400 transmission electron microscope at an operating voltage of 80 kV and magnification of 4000–8000.

### Data analysis

The capsid motion images were processed using non-local means algorithm [64] to reduce their noise. The capsids were detected and their coordinates extracted using TrackMate (version 6.0.1) [65] plugin in Fiji [66]. The capsid spots were detected using LoG detector with spot size of 4 pixels (520 nm). Because at later time points the number of capsids can be very high, a spot quality threshold of 10 was used to filter out spots that were dimmer or not close to the specified size (and therefore likely resulting from capsids located in out-of-focus regions). The spots were connected using simple LAP tracker with a linking and gap-closing max distance of 4 pixels and a gap-closing maximum frame gap of 2 frames. The mean number of nuclear tracks that were analyzed was 1100 ± 200 at 4 hpi, 2600 ± 300 at 8 hpi and 3600 ± 400 at 12 hpi. The mean nuclear track length was 1.12 ± 0.06 s, 0.90 ± 0.04 s and 0.85 ± 0.07 s, and the number of detected capsids per image was 35 ± 5, 66 ± 7 and 72 ± 7 at 4, 8 and 12 hpi, respectively. The mean nearest neighbor distance between two tracked capsids in the first imaged frame was 1.5 ± 0.1 μm at 4 hpi, 1.35 ± 0.08 μm at 8 hpi and 1.21 ± 0.05 μm at 12 hpi, which are well above the used linking distance of 4 pixels (520 nm) for capsid spots, showing that tracking individual spots is reliable. It was also verified afterwards that the number of detected jumps corresponding to the maximum linking distance was already very low.

The distance profile of Hoechst was calculated by sorting the Euclidean distance values of nuclear pixels to the nuclear envelope into a bins of two pixels (260 nm) wide and averaging the pixel intensities of each bin. The chromatin and interchromatin regions were segmented using Otsu’s algorithm [67] and the segmentation of nuclei was achieved by filling any holes left behind from chromatin segmentation step separately in each confocal layer.

The mean squared displacement of particles in chromatin and interchromatin regions was calculated so that tracks that started at least two pixels away from the chromatin/interchromatin interface were taken into account. If a particle arrived within 2 pixels from the border, the analysis of that particle was terminated. The distance limit of two pixels was introduced to take into account possible inaccuracies in the segmentation of the regions. The equation for anomalous diffusion was fitted for each MSD(t) in the time range of 0–1 s. Sometimes the curve saturated before one second, and in those cases the fitting was done to the nonsaturated part of the curve.

The crossing frequency between chromatin and chromatin-empty regions was evaluated by taking into account all capsids that were at least two pixels but not more than four pixels away from the border between the regions and then calculating the probability that those capsids ended up on the other side of the border during their tracks. The lower limit was again introduced to be sure that the capsid was at the denoted side of the interface. The upper limit was introduced because when VRCs grow at later time points, capsids are on average located further away from the chromatin and are less likely to cross into chromatin regions. This is considered by examining only particles that are located close to the border between chromatin and chromatin-empty regions. The fraction of immobile capsids was analyzed by taking the first frame of the time series and comparing the number of capsids that did not move significantly to the total number of capsids. The capsid was marked as immobile if the capsid track was at least 5 seconds long and it did not move during the track. A small movement of three pixels (490 nm) or less was allowed to take into account small fluctuations in cellular and chromatin positions as well as detection inaccuracies.

All the values were calculated as the mean ± the standard deviation of the mean (SEM) calculated over all the cells unless otherwise stated. The data used in the statistical calculations are given in S1 Data.

## Supporting information

S1 Fig Viral replication compartment fills the chromatin-empty regions. Confocal microscopy images of chromatin and VRCs at 4, 8 and 12 hpi visualized using Hoechst 33342 (gray) and antibody against viral ICP8 (magenta). The scale bar represents 5 μm.
(TIF)

S2 Fig Chromatin border is localized at the nuclear lamina. Confocal microscopy images of Hoechst and lamin B antibody labeled cell nuclei. Nuclear borders obtained via automatic segmentation using minimum cross entropy segmentation are shown (chromatin border red, lamin border green). The generated borders match well, but cytoplasmic accumulation of lamin B staining during infection makes segmentation with lamin staining sometimes unreliable (see the bottom nuclei imaged at 12 hpi). The scale bar represents 5 μm.
(TIF)

S3 Fig Hoechst and fluorescent H2B label chromatin regions similarly. Example images showing labeling of chromatin using Hoechst and fluorescent histone protein H2B. The Dice coefficient, defined as the size of the overlapping region of the segmentations divided by the mean size of the regions was 0.79, 0.83 and 0.88 at 4, 8 and 12 hpi, respectively. For each time point *N* = 10. The scale bar represents 5 μm.
(TIF)

S4 Fig Anomalous diffusion fits in the nucleus. An example fit of equation *MSD* = 4*Dt*^*α*^ in chromatin and chromatin-empty regions of a cell infected 8 h prior to measurement.
(TIF)

S1 Movie A time series of capsid movement in the cell. The motion of capsids (green) shown overlaid on the chromatin structure of the cell (gray).
(AVI)

S1 Data Data values used in the statistical analyses. (XLSX)
