# Supplementary figures and images for: Infection-induced chromatin modifications facilitate translocation of herpes simplex virus capsids to the inner nuclear membrane
Source: PLoS Pathog. 2021 Dec 15;17(12):e1010132. doi: 10.1371/journal.ppat.1010132 (PMC8673650; doi:10.1371/journal.ppat.1010132)

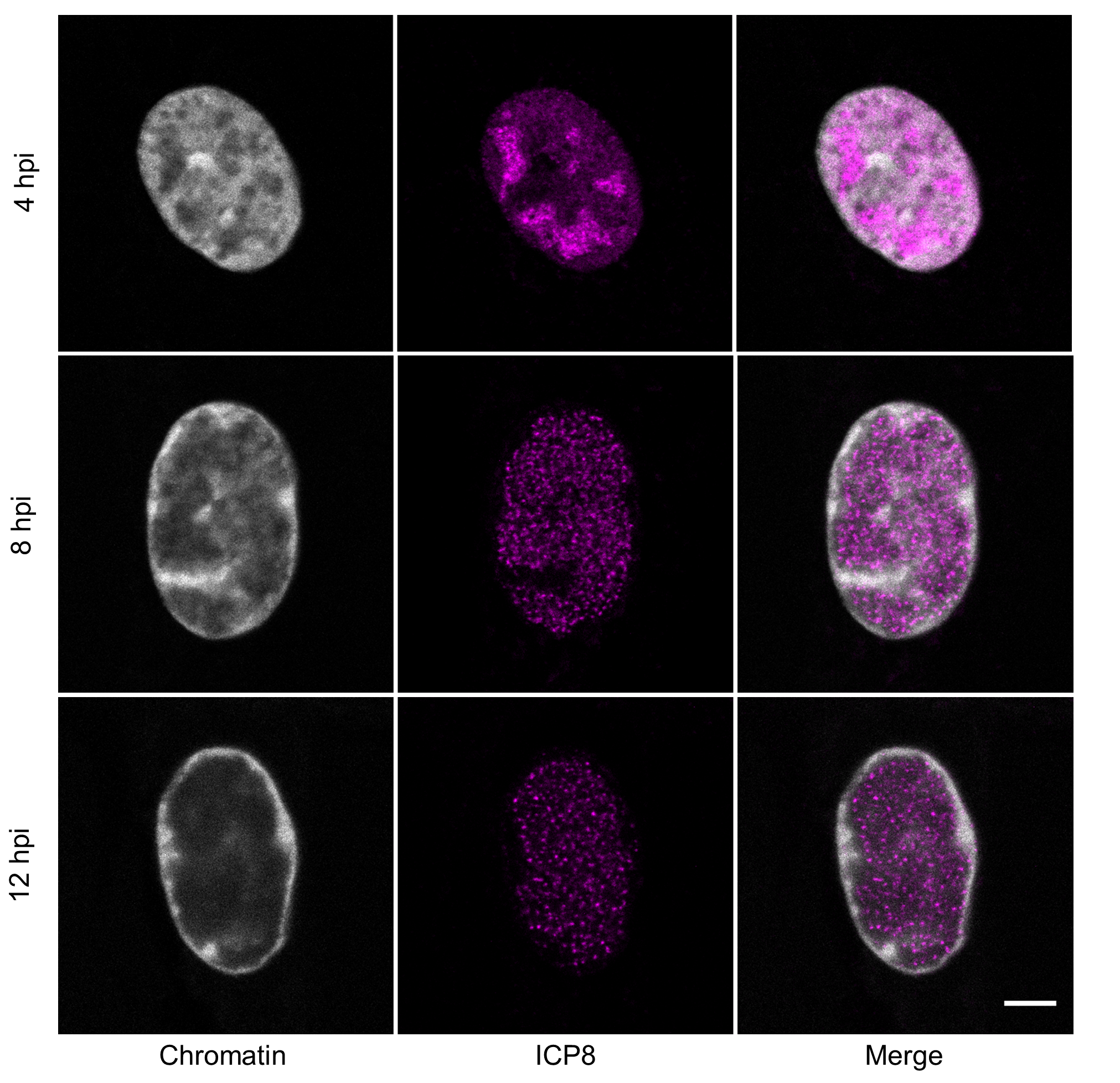

Supplement: S1 Fig — Confocal microscopy images of chromatin and VRCs at 4, 8 and 12 hpi visualized using Hoechst 33342 (gray) and antibody against viral ICP8 (magenta). The scale bar represents 5 μm. (TIF) [file ppat.1010132.s001.tif]

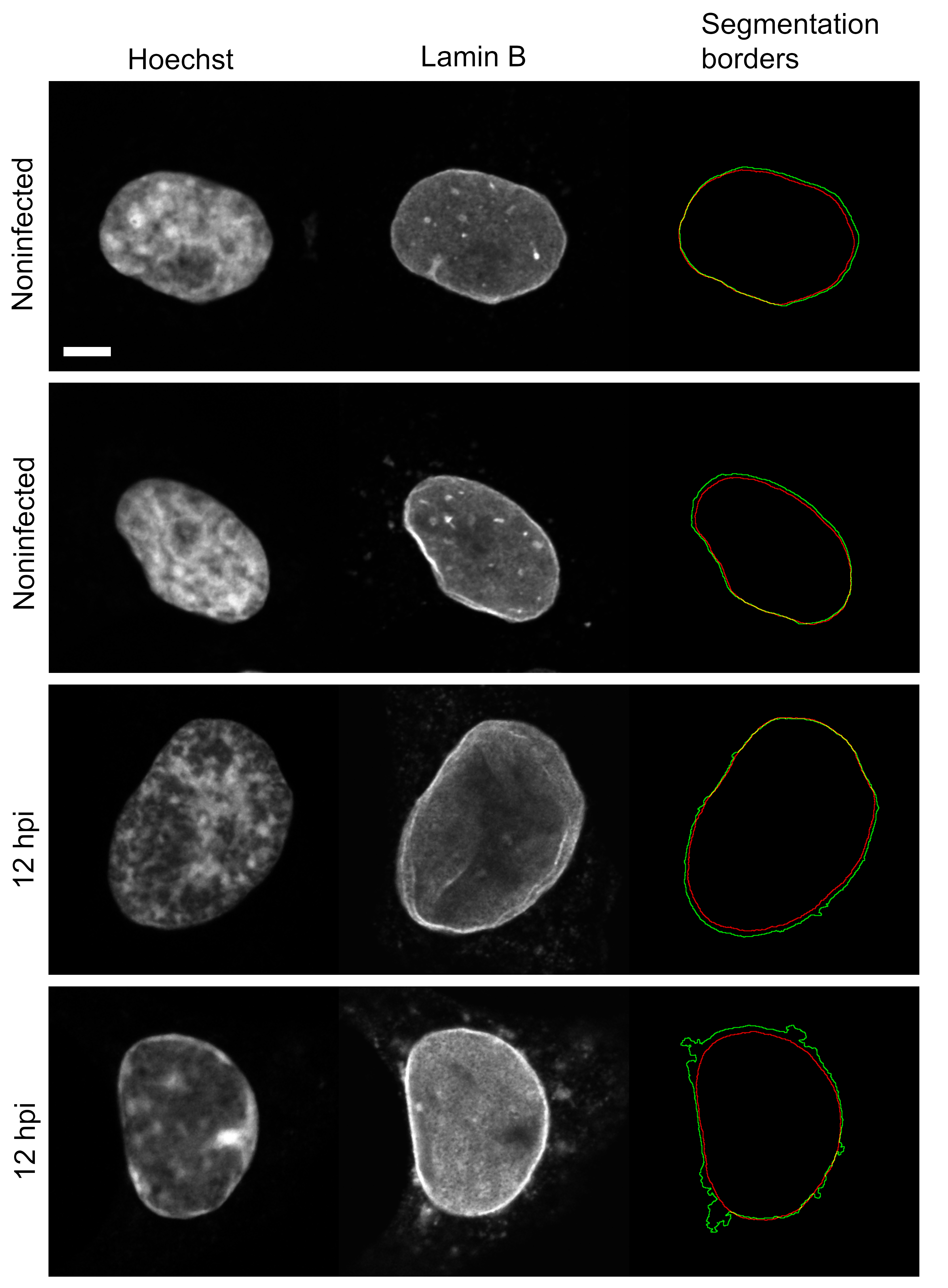

Supplement: S2 Fig — Confocal microscopy images of Hoechst and lamin B antibody labeled cell nuclei. Nuclear borders obtained via automatic segmentation using minimum cross entropy segmentation are shown (chromatin border red, lamin border green). The generated borders match well, but cytoplasmic accumulation of lamin B staining during infection makes segmentation with lamin staining sometimes unreliable (see the bottom nuclei imaged at 12 hpi). The scale bar represents 5 μm. (TIF) [file ppat.1010132.s002.tif]
